# Factors Associated With Visual Acuity in Non-arteritic Ischemic Optic Neuropathy Patients: A Five-Year Cross-Sectional Study

**DOI:** 10.7759/cureus.29156

**Published:** 2022-09-14

**Authors:** Parinee Kemchoknatee, Chotika Singhakul, Duanghathai Tangon, Thansit Srisombut

**Affiliations:** 1 Department of Ophthalmology, Rangsit University, Rajavithi Hospital, Bangkok, THA

**Keywords:** hypertension, diabetes mellitus, systemic vascular disease, visual field, visual acuity, visual outcome, non-arteric ischemic optic neuropathy

## Abstract

Background and objective

Non-arteritic ischemic optic neuropathy (NAION) is a common cause of optic neuropathy in elderly patients. Currently, there is no definitive treatment for this condition, and the factors influencing visual outcomes have not yet been conclusively identified. In this study, we aimed to evaluate factors that affect visual outcomes and those that are predictors of the development of NAION in a Thai population.

Methods

All patients diagnosed with NAION at the Rajavithi Hospital between January 1, 2016, and December 31, 2020, were retrospectively reviewed to evaluate the improvement in their best-corrected visual acuity (BCVA) and determine the factors that are predictive of visual outcomes.

Results

The 80 patients reviewed were predominantly male (55%) with a mean age of 55.8 ±9.89 years. Their most common comorbidities were dyslipidemia (DLP) (67.5%), diabetes mellitus (DM) (61.3%), and hypertension (HT) (48.8%). At the 12-week follow-up visit, there was a significant improvement of at least 0.2 logarithm of the minimum angle of resolution (logMAR) in BCVA (p=0.001). A significantly greater percentage of patients with higher age, DM, and HT was observed in the unfavorable visual recovery (UVR) group (p=0.002, p=0.001, and p=0.005 respectively). In contrast, neither baseline visual acuity nor cup-to-disc ratio (CDR) affected the result of visual recovery (p=0.275 and p=0.076, respectively). In multivariate logistic analysis, older age increased the odds of worse visual recovery [odds ratio (OR): 4.014; 95% CI: 1.038-15.515; p=0.044], as did having DM (OR: 3.809; 95% CI: 1.168-12.421; p=0.027), and HT (OR: 4.577; 95% CI: 1.491-14.049; p=0.008).

Conclusions

None of the baseline visual status parameters (visual acuity, CDR, or visual field defect) was able to determine the outcome of visual recovery at 12 weeks in our NAION patients. Regarding systemic vascular diseases, diabetes and HT are significant risk factors and also predictors of poor visual improvement in Thai populations. NAION patients who are elderly or have vascular diseases such as DM or HT should be closely followed up and advised about the likelihood of having inferior visual recovery.

## Introduction

Non-arteritic ischemic optic neuropathy (NAION) is the most common cause of optic neuropathy in elderly persons. Patients with NAION typically present with painless visual loss, impaired color vision, disc swelling, presence of relative afferent papillary defect (RAPD), and optic-nerve-related visual field defect [1]. Previous researchers have found systemic vascular diseases, such as type 2 diabetes mellitus (T2DM), hypertension (HT), dyslipidemia (DLP), and ischemic heart disease (IHD), to be risk factors for developing NAION [2-5]. Hayreh et al. proposed that these vascular diseases did not affect visual outcomes [6]; however, Sharma et al. observed that patients with IHD carried a higher risk of having worse final visual acuity [7]. In addition, Dattilo et al. suggested that having multiple vascular diseases rendered patients in western countries prone to having a worse final vision [8]. With regard to Asian populations, there is insufficient data to determine whether vascular risk factors influence visual results.

The purpose of this study was to evaluate factors influencing visual outcomes and identify factors that predispose Thai individuals to develop NAION.

## Materials and methods

Study design and data collection

In this retrospective cohort study, the electronic medical records of 124 patients diagnosed with NAION between January 1, 2016, and December 31, 2020, were retrospectively reviewed. This research was approved by the Rajavithi Hospital Ethics Committee and it followed the tenets of the Declaration of Helsinki. All participants gave written informed consent before participating in the study.

The inclusion criteria were NAION patients diagnosed during the study period by ophthalmologists in our center based on the parameters used in the research by Sharma et al. [7], which were as follows: acute painless visual loss with the evidence of optic neuropathy including the presence of RAPD; presenting a swelling disc morphology within one month; impaired color vision test; or nerve fiber layer defect on a visual field test.

The exclusion criteria were as follows: (1) patients with other optic neuropathies such as optic neuritis, anterior arteritic ischemic optic neuropathy (AION), or compressive optic neuropathy; (2) those with other retinal diseases such as proliferative diabetic retinopathy, macular edema; (3) patients having other eye diseases affecting visual acuity; (4) follow-up time of less than three months; (5) patients having an onset duration greater than 30 days; (6) incomplete medical record data; (7) optic neuropathy with a history of previous intraocular surgery within one month. Risk factors associated with systemic vascular disease were identified based on a review of the literature: T2DM, arterial HT, obstructive sleep apnea (OSA), DLP, IHD, and history of cerebrovascular disease. All related comorbidities were recorded by internists at our institute. Diagnostic criteria were based on the latest international guidelines [9-11].

All patients underwent complete ophthalmic evaluations at the initial visit, one week, four weeks, and 12 weeks after onset duration, including best-corrected visual acuity (BCVA), color vision tested by Ishihara color plate, pupillary defect, fundus, and optic disc examination. The visual field was tested with Humphrey Field Analyzer HFA II 750 (Carl Zeiss Meditec Inc, Dublin, CA) using a 24-2 threshold program with the Swedish Interactive Threshold Algorithm (SITA) Faster strategy. In our series, the main criterion of favorable visual recovery (FVR) was defined as better than or at least 0.2 logarithm of the minimum angle of resolution (logMAR) in BCVA at the 12-week visit compared to that at the presentation. Unfavorable visual recovery (UVR) was classified as either progressive (BCVA worsened by more than 0.2 logMAR) or stable (BCVA worsened/improved by less than 0.2 logMAR) at the 12-week visit compared to that at the presentation. Significant improvement in the visual field was defined as at least or better than 2 decibels (dB) in the mean deviation (MD) of the visual field at the 12-week visit compared to the presentation.

Ethical approval

The study was approved by the Rajavithi Hospital Research Ethics Committee (EC number: 020/2565). All the patients provided written informed consent before data were collected.

Statistical analysis

Shapiro-Wilk test and histogram were employed to test the normality of the distribution of the data. All affected eyes were included in the study for analysis. Continuous data were expressed as means and standard deviation in normally distributed data, while median and interquartile range (IQR) were used for non-normally distributed data. Categorical variables including gender, presence of RAPD, smoking status, and disc morphology were analyzed with chi-square or Fisher's exact test. Paired t-tests were employed for comparing the differences in baseline visual acuity and that at each visit (BCVA was converted to logMAR for statistical purposes) and visual field, the difference in dB between the initial and each subsequent visit, was analyzed by Mann-Whitney U test. Binary logistic regression was carefully performed based on previous relevant research to identify the factors influencing the achievement of a better final visual outcome. The variables considered potentially significant with a p-value less than 0.2 were considered in the multivariate binary logistic regression analysis. Statistical significance was set at p-value <0.05. All statistical analyses were performed with SPSS Statistics version 25 (IBM Corp., Armonk, NY).

## Results

A total of 124 patients with NAION (130 eyes) were enrolled in our study. Forty-four patients were excluded due to not fulfilling our inclusion criteria: 15 had a follow-up time of less than three months; a further 14 had incomplete medical records; five had other optic neuropathy diseases; eight had a duration after the onset of greater than 30 days; two had pale disc morphology at initial assessment. In the case of sequential or concurrent NAION, only the first eye affected was included in our analysis. In our series, six patients presented with sequential NAION, and none of our patients were reported as concurrent or recurrent NAION.

A total of 80 patients with NAION (80 eyes) were recruited in the present study. As shown in Table 1, the mean age ±SD of the cohort was 55.8 ±9.89 years. There was a significant difference in mean age between the FVR and UVR groups (p=0.002). More than half were male and had a small CDR. With regard to comorbidities, DLP, T2DM, HT, IHD, and cerebrovascular disease were observed as shown (Table 1).

**Table 1:** Baseline characteristics of NAION patients at initial diagnosis *Defined as CDR <0.3. ^†^Fisher's exact test NAION: non-arteritic ischemic optic neuropathy; FVR: favorable visual recovery; UVR: unfavorable visual recovery; SD: standard deviation; CDR: cup-to-disc ratio; T2DM: type 2 diabetes mellitus; HT: hypertension; DLP: dyslipidemia; IHD: ischemic heart disease; CVA: cerebral vascular accident; OSA: obstructive sleep apnea

Characteristic	Total eyes (80)	FVR (31)	UVR (49)	P-value
Mean age, years, mean ±SD	55.8 ±9.89	51.7 ±9.06	58.5 ±9.56	0.002
Old age (≥50 years), n (%)	61 (76.3)	17 (54.8)	44 (89.8)	<0.001
Female sex, n (%)	36 (45)	16 (51.6)	20 (40.8)	0.344
Tobacco use, n (%)	5 (6.3)	1 (3.2)	4 (8.2)	0.644^†^
Small CDR*, n (%)	45 (56.3)	16 (51.6)	29 (59.2)	0.506
Comorbid diseases, n (%)				
T2DM	49 (61.3)	12 (38.7)	37 (75.5)	0.001
HT	39 (48.8)	9 (29)	30 (61.2)	0.005
DLP	54 (67.5)	20 (64.5)	34 (69.4)	0.65
IHD	5 (6.3)	2 (6.5)	3 (6.1)	1^†^
CVA	2 (2.5)	0 (4.1)	2 (4.1)	0.519^†^
OSA	0			
Duration after onset, days, mean ±SD	22.6 ±8.30	21.9 ±9.04	23 ±7.85	0.572

The comparison of baseline characteristics between the FVR and UVR groups showed that the UVR group had more patients with older age, DM, and arterial HT, and the difference was statistically significant (p<0.001, p=0.001, p=0.005 respectively). However, there were no statistically significant differences between the two groups in gender, tobacco use, duration after onset, and the presence of the other three comorbid diseases, namely DLP, IHD, and cerebrovascular disease (Table 1).

As shown in Table 2, the mean baseline BCVA of all affected cases was 1.32 ±0.92 logMAR. Just over half of the affected eyes were right-side ones. The mean CDR of all patients was 0.32 ±0.08. All cases had a swelling disc appearance at the baseline visit, and almost half (47.5%) presented with peripapillary splinter hemorrhage at the initial visit. The mean visual field defect was -20.44 ±1.44 dB. In our series, none of the visual parameters, including BCVA, CDR, color vision loss, and visual field defect, significantly varied between the two groups in terms of visual recovery outcomes.

**Table 2:** Baseline visual parameters at presentation* *In cases of bilateral NAION, we analyzed the first eye affected NAION: non-arteritic ischemic optic neuropathy; FVR: favorable visual recovery; UVR: unfavorable visual recovery; BCVA: best-corrected visual acuity; SD: standard deviation; CDR: cup-to-disc ratio

Characteristic	Total eyes (80)	FVR (31)	UVR (49)	P-value
BCVA at baseline, mean ±SD	1.32 ±0.92	1.46 ±0.89	1.23 ±0.93	0.275
Dyschromatopsia, n (%)	56 (70)	24 (77.4)	32 (65.3)	0.249
Afferent pupillary defect, n (%)	54 (67.5)	24 (77.4)	30 (61.2)	0.132
CDR, mean ±SD	0.32 ±0.08	0.3 ±0.06	0.33 ±0.08	0.076
Optic disc morphology, n (%)				
Swollen, n (%)	80 (100)			
Peripapillary splinter hemorrhage, n (%)	38 (47.5)	18 (58.1)	20 (40.8)	0.132
Humphrey visual field, dB, mean ±SD	-20.44 ±1.44	-22.01 ±13.47	-19.44 ±12.51	0.804

As displayed in Table 3, 25%, 35%, and 38.8% of patients had an improved logMAR of at least 0.2 at their one-week, four-week, and 12-week visits respectively when compared with the baseline values. At the one-week visit, we found no significant visual improvement (p=0.079). A significant improvement in BCVA was noted at the four-week and 12-week follow-up visits compared to the initial presentation (p=0.039, p=0.001 respectively).

**Table 3:** Outcomes in BCVA at one-week, four-week, and 12-week follow-ups ^†^Based on comparison with the values at the baseline visit BCVA: best-corrected visual acuity; SD: standard deviation: logMAR: logarithm of the minimum angle of resolution

Characteristic	Total eyes (80)	P-value
BCVA at 1 week		0.079^†^
Mean ±SD	1.21 ±0.89	
Improved (0.2 logMAR), n (%)	20 (25)	
Difference between baseline and at 1 week (mean ±SD)	0.11 ±0.56	
BCVA at 4 weeks		0.039^†^
Mean ±SD	1.18 ±0.95	
Improved (0.2 logMAR), n (%)	28 (35)	
Difference between baseline and at 4 weeks (mean ±SD)	0.14 ±0.59	
BCVA at 12 weeks		0.001^†^
Mean ±SD	1.06 ±0.92	
Improved (0.2 logMAR), n (%)	31 (38.8)	
Difference between baseline and at 12 weeks (mean ±SD)	0.26 ±0.66	

The data of the 80 patients underwent univariate and multivariate logistic regression analysis as shown in Table 4. In univariate analysis, older age (≥50 years), T2DM, and HT were statistically significant risk factors. After multivariate analysis, these three variables remained statistically significant: old age (≥50 years) decreased the chance of achievement of improved BCVA (by at least 0.2 logMAR) at 12-week follow-up compared to baseline [odds ratio (OR): 4.014; 95% CI: 1.038-15.515; p=0.044]. Similarly, DM was found to lower the chance of successful outcomes (OR: 3.809; 95% CI: 1.168-12.421; p=0.027). Also, HT had a strong negative impact on the final BCVA (OR: 4.577; 95% CI: 1.491-14.049; p=0.008).

**Table 4:** Comparison of baseline characteristics and disease outcomes *Patients aged ≥50 years. **Cut-off point of 20/200 (1 logMAR). ***Defined as CDR <0.3 CI: confidence interval; VA: visual acuity; CDR: cup-to-disc ratio; T2DM: type 2 diabetes mellitus; HT: hypertension; DLP: dyslipidemia; IHD: ischemic heart disease

	Univariate analysis			Multivariate analysis			
Variable	Odds ratio	95% CI	P-value		Odds ratio	95% CI	P-value
Old age*	7.247	2.262-23.221	0.001		4.014	1.038-15.515	0.044
Female	0.647	0.261-1.6	0.345		-	-	-
Smoker	2.667	0.284-25.036	0.391		-	-	-
Poor baseline VA**	0.658	0.264-1.642	0.369		-	-	-
Small CDR***	1.359	0.549-3.363	0.507		-	-	-
T2DM	4.882	1.846-12.914	0.001		3.809	1.168-12.421	0.027
HT	3.860	1.47-10.133	0.006		4.577	1.491-14.049	0.008
DLP	1.247	0.480-3.237	0.651		-	-	-
IHD	0.946	0.149-6.006	0.953		-	-	-

Both univariable and multivariable logistic regression revealed that the variables that did not influence the achievement of improved BCVA between the initial visit and the final 12-week follow-up period were gender, tobacco use, poor baseline visual acuity, small CDR, DLP, and IHD.

Regarding visual field defect, 34.6%, 38.5%, and 50% of patients had an improvement of at least 2 dB at one-, four-, and 12-week follow-up visits respectively when compared to the baseline values (Table 5; data of only 26 patients with a complete visual field assessment are shown)

**Table 5:** Outcomes in VF at one-week, four-week, and 12-week follow-ups* *Only 26 eyes with complete visual field data are shown. The comparison was made with the baseline visual field

Characteristic	Total (26)
VF at 1 week	
Improved (0.2 dB), n (%)	9 (34.6)
VF at 4 weeks	
Improved (0.2 dB), n (%)	10 (38.5)
VF at 12 weeks	
Improved (0.2 dB), n (%)	13 (50)

## Discussion

The present study found some common comorbidities in NAION patients, including DLP, T2DM, and HT. In addition, we found that older age (≥50 years), DM, and HT were identified as significant risk factors for worse visual recovery in the Thai population we studied.

In a previous study of a western population, Scherer et al. revealed that younger age groups had better visual outcomes [12]. Similarly, Preechawat et al. reported that young patients (<50 years) had a higher proportion of final VA of better than 20/63, and only 13% presented with a VA of worse than 20/200 [13]. However, Sun et al. reported contradictory results, revealing no association between patient age and final VA [14]. There have been contrasting results in studies from other regions. Lin et al. reported no age-related difference in visual recovery (≥0.2 logMAR) [15], while Behbehani et al. disagreed, finding a statistically significant, almost three-fold greater achievement of VA of better than 20/40 at three months in younger age groups (<50 years); they attributed these results to the fact that there were fewer comorbidities and better baseline visual status in the young [16]. The present study observed that older age (≥50 years) significantly increased the risk of failing to achieve good visual recovery (at least 0.2 logMAR) by four times. Our finding was supported by Sharma et al. who revealed that being older (≥50 years) increased the odds of a worse final vision (VA>20/200) [7]. We agree with Behbehani et al. in that the findings in visual outcomes in older age might be explained by a higher proportion of systemic vascular diseases. We propose that a larger sample of NAION cases is necessary to address these associations in Thai populations.

Hayreh et al. and the IONDT group revealed no association between systemic vascular diseases (DM, HT, or DLP) and final visual outcomes [6,17]. In addition, Sharma et al. reported that having DM did not affect final visual results [7]. Similarly, Cullen et al. showed that none of these vascular diseases (DM, HT, or DLP) affected the final visual prognosis in Singapore [18]. In contrast, a recent study on a western population by Dattilo et al. found a trend towards poor visual function (worse than hand motion) in patients with multiple systemic vascular diseases and older age [8]. In an Indian study, Patil et al. found an association between multiple vascular diseases and very poor final visual outcomes; however, their results were not statistically significant (p=0.09) [19]. Interestingly, our series observed that having T2DM increased the risk of poor visual recovery at 12 weeks by almost four times and that HT increased the odds ratio by 4.5-fold after adjusting for all covariable factors in multivariate logistic regression analysis. Our results are in line with those of a Kuwait study and of Eagling et al. who suggested that patients without systemic vascular disease gained better final visual acuity [16,20]. According to Hayreh et al., the non-diabetic group in their study experienced a disc swelling resolution time that was three times shorter than that of the diabetic group (p=0.003) [21]. However, the researchers noted that the early resolution of disc swelling may not reflect a better visual result. Unfortunately, we could not assess this aspect in our report because of its retrospective nature.

The overall improvement in visual field defect in dB at the last visit (12 weeks after the initial visit) was 50% (13 out of 26 patients). There was a stable improvement in the visual field during the follow-up period compared to the findings by Chang et al. who observed a trend of significant improvement at one-to-three-month follow-up after the onset of the disease in Korean patients [22]. We propose some explanations as follows: the first entails the different methods of visual field assessments between ours and that of Chang et al.; while ours measured the difference of mean deviation, Chang et al. employed Goldman Perimetry evaluation [22]. Secondly, the small number of patients may prevent drawing a definitive comparison. Several previous researchers found no association between systemic vascular diseases and visual field outcomes [6,12,23]. Unfortunately, we could not study predictors of visual field outcomes in our logistic model due to the small number of patients with a complete visual field examination. We believe that it would be worth studying whether or not having diabetes or HT affected final visual field results in our Thai patients. We propose that the influence of DM, HT, or other vascular diseases should be prospectively studied in terms of visual outcomes of VA and VF in a larger sample size in our population.

We found that gender, initial VA, small CDR, DLP, and IHD had no association with final visual recovery in the present study. Lee et al. proposed that the male gender increased the risk of developing NAION by 32% [24]. Dattilo et al. observed a higher proportion of males with initial VA worse than hand motion in western populations [8]. Conversely, Hayreh et al. found no gender-related predominance in terms of outcomes of VA at one-year follow-up [6]. Although Lin et al. observed a tendency for males to be prone to attaining worse visual outcomes in a Chinese population, their results did not reach statistical significance [15]. In this series, although we found a male predominance in patients with NAION, being male was not a significant predictive risk factor for worse outcomes. Our results have been supported by Bernstein et al. who reported no advantage of estrogen therapy in the rodent NAION model [25]. Similarly, Nuzzi et al. proposed that estrogen provided no potentially protective effect against NAION [26]. Previous researchers have suggested that there is no correlation between initial VA and final vision and this is in agreement with our results [6,15].

Small CDR was observed in 56% of our participants. It is widely hypothesized that swelling optic nerve fibers in a small crowded disc are among the predisposing factors of NAION; however, the relationship between disc ratio and disc size with respect to final vision is still a matter of controversy [1,21]. Previous researchers have found no association between disc ratio and visual outcomes of the disease [7,16]. Likewise, Gonzalez et al. reported that small CDR is an important risk factor for developing the disease; however, there was no association with visual prognosis [27]. Our findings are in agreement with previous studies that observed no correlation between disc ratio and visual recovery [7,16].

Kim et al., in a study of a Korean population, revealed that hypercholesterolemia increased the risk of developing NAION by five times [28]. Our study found that it was the most common co-existing vascular disease (67.5%) in our cohort of a Thai population, but we found that it did not increase the risk of worse visual outcomes at the 12-week follow-up; this is in accordance with the findings of previous studies [6,18]. Likewise, IHD was not found to carry a higher danger of achieving worse outcomes. Although our findings contrasted with those of a previous report which noted that IHD raised the risk of having a final VA of worse than 20/200 almost six-fold [7], other studies found no relationship between this disease and visual outcomes [6,17]. We believe that the small number of cases with IHD in our series (five patients) may have weakened the power of analysis. Tobacco use was not identified as a risk factor for worse visual recovery in the current study.

To the best of our knowledge, this is the first study to reveal that older age and some systemic vascular diseases (such as DM and HT) affected visual outcomes in a Thai population. Some limitations of our research should be noted. Firstly, we could not assess factors affecting the outcomes of visual field defects due to the small number of patients (26 patients). Second, some risk factors may have been underdiagnosed; for example, no cases of the use of phosphodiesterase 5 inhibitors were reported, and none of our patients had OSA. Third, some visual parameters or factors, such as nocturnal hypotension or the change of optic nerve in optical coherence tomography (OCT), could not be assessed due to the retrospective design of our research.

## Conclusions

None of the baseline visual status parameters (visual acuity, CDR, or visual field) was able to reflect the outcomes of visual recovery at 12 weeks in our NAION patients. Regarding systemic vascular diseases, diabetes and HT are significant risk factors and also predictors of poor visual improvement in patients with NAION in our cohort of Thai patients. NAION patients who are older or have those vascular diseases (such as DM or HT) should be closely followed up and counseled about the possibility of having poor visual recovery.
